# Genetic profiling of azoospermic men to identify the etiology and predict reproductive potential

**DOI:** 10.1007/s10815-024-03045-5

**Published:** 2024-02-26

**Authors:** Stephanie Cheung, Lily Ng, Philip Xie, Olena Kocur, Rony Elias, Peter Schlegel, Zev Rosenwaks, Gianpiero D. Palermo

**Affiliations:** 1 The Ronald O. Perelman and Claudia Cohen Center for Reproductive Medicine, Weill Cornell Medicine, 1305 York Avenue, Y720, New York, NY 10021 USA; 2 Department of Urology, James Buchanan Brady Foundation and Cornell Reproductive Medicine Institute, Weill Cornell Medicine, New York, NY USA

**Keywords:** Azoospermia, Genomics, ICSI, Next generation sequencing, Testicular sperm retrieval

## Abstract

**Purpose:**

To identify germline mutations related to azoospermia etiology and reproductive potential of surgically retrieved spermatozoa, and to investigate the feasibility of predicting seminiferous tubule function of nonobstructive azoospermic men by transcriptomic profiling of ejaculates.

**Materials and methods:**

Sperm specimens were obtained from 30 men (38.4 ± 6 years) undergoing epididymal sperm aspiration for obstructive azoospermia (OA, *n* = 19) acquired by vasectomy, or testicular biopsy for nonobstructive azoospermia (NOA, *n* = 11). To evaluate for a correlation with azoospermia etiology, DNAseq was performed on surgically retrieved spermatozoa, and cell-free RNAseq on seminal fluid (*n* = 23) was performed to predict spermatogenesis in the seminiferous tubule.

**Results:**

Overall, surgically retrieved sperm aneuploidy rates were 1.7% and 1.8% among OA and NOA cohorts, respectively. OA men carried housekeeping-related gene mutations, while NOA men displayed mutations on genes involved in crucial spermiogenic functions (*AP1S2*, *AP1G2*, *APOE*). We categorized couples within each cohort according to ICSI clinical outcomes to investigate genetic causes that may affect reproductive potential. All OA-fertile men (*n* = 9) carried mutations in *ZNF749* (sperm production), whereas OA-infertile men (*n* = 10) harbored mutations in *PRB1*, which is essential for DNA replication. NOA-fertile men (*n* = 8) carried mutations in *MPIG6B* (stem cell lineage differentiation), whereas NOA-infertile individuals (*n* = 3) harbored mutations in genes involved in spermato/spermio-genesis (*ADAM29*, *SPATA31E1*, *MAK*, *POLG*, *IFT43*, *ATG9B*) and early embryonic development (*MBD5*, *CCAR1*, *PMEPA1*, *POLK*, *REC8*, *REPIN1*, *MAPRE3*, *ARL4C*). Transcriptomic assessment of cell-free RNAs in seminal fluid from NOA men allowed the prediction of residual spermatogenic foci.

**Conclusions:**

Sperm genome profiling provides invaluable information on azoospermia etiology and identifies gene-related mechanistic links to reproductive performance. Moreover, RNAseq assessment of seminal fluid from NOA men can help predict sperm retrieval during testicular biopsies.

**Supplementary Information:**

The online version contains supplementary material available at 10.1007/s10815-024-03045-5.

## Introduction

Male infertility accounts for approximately half of the causes of the inability to reproduce among infertile couples [1]. Fortunately, the introduction of ICSI has enhanced the treatment of even the most extreme forms of male factor infertility [2]. Among the different etiologies of male reproductive failure, the most challenging is azoospermia, which accounts for approximately 30% of all cases [3]. The most puzzling form of azoospermia is the testicular type [4]. While it can be attributed to genetic disorders, developmental abnormalities, hormonal imbalances, or consequent exposure to chemotherapy or radiation, the remaining cases are classified as idiopathic [5]. To define azoospermia, a detailed assessment of the patient’s medical history is performed, and various evaluations are carried out [6]. In cases of obstructive azoospermia (OA), assessment of the ejaculate does not yield any spermatozoa, whereas in cases of non-obstructive azoospermia (NOA), the ejaculate may inconsistently yield spermatozoa, representing an additional challenge that often requires an extensive search to identify sperm cells [2].

Once surgical approach is considered, chances of successful sperm retrieval in OA cases is likely to be >99%, while cases with testicular failure can be very unpredictable [2, 7]. Furthermore, if spermatozoa are identified in NOA cases, there also lies the ambiguity of whether azoospermia is due to primary testicular failure or depends on secondary environmental factors [8]. Therefore, it is crucial to characterize the genotypes of azoospermic individuals with impaired germ cell replication and gamete differentiation [9].

Genetic testing has become increasingly prevalent in male infertility screening and includes testing for whole-chromosomal structural aberrations, partial chromosomal defects, and monogenic diseases [10]. In men with azoospermia, genetic screening has revealed a higher incidence of constitutional karyotypic abnormalities [11]. Detection of Y-microdeletions can also identify genetic relationships of impaired spermatogenesis, which would not only remarkably limit reproductive competence, but would also be passed on to male offspring once assisted reproductive technologies (ART) are used [12]. Over the years, the application of genetic testing in azoospermic men has evolved, but it continues to primarily target the individual’s somatic tissue, and at times, a discrepancy between de-novo mutations on the germline vis-à-vis the somatic cells has been reported [13].

To date, genetic assessments have been aimed at highlighting the etiology of compromised sperm production [10]; however, the influence of genomic aberrations on the reproductive competence of surgically retrieved gametes remains relatively unknown.

Testicular spermatozoa have long been considered to be plagued by more chromosomal abnormalities than their ejaculated counterparts and were therefore perceived as more likely to generate aneuploid embryos [14–16]. This has recently been refuted [17], and subsequent reports have evidenced that the chromosomal and developmental characteristics of children born from spermatozoa retrieved from the seminiferous tubule are normal [17, 18]. This acquired knowledge on the safety of these gametes reiterates the relevance of understanding the etiology of the most common and concerning form of azoospermia, and reinforces interest in assessing the embryo developmental competence of these spermatozoa. Therefore, genetic assessment of the germline in relation to ART outcomes is necessary.

A pressing need has emerged for the treatment of NOA individuals, prompted by the unpredictability of sperm retrieval during testicular biopsy. This has recently been investigated by identifying rare polymorphisms, differentially expressed genes (DEGs), and the underlying DNA methylation mechanisms that affect sperm development [19, 20]. While most of this information was obtained from retrospective analyses of somatic cells, identification of genomic factors capable of non-invasively predicting successful retrieval of spermatozoa during testicular biopsy is of utmost importance to help streamline the clinical management of men with NOA.

In this study, we performed whole exome sequencing (WES) of the genome of spermatozoa retrieved surgically from azoospermic men to delineate germline mutations that may reveal the etiology of their spermatogenic failure and simultaneously elucidate their specific embryo developmental competence. In a subanalysis, we proposed a non-invasive method to identify testicular transcriptomes from cell-free RNAs in seminal fluid from NOA men to predict spermatozoa retrieval at testicular biopsy.

## Materials and methods

### Inclusion criteria and study design

**Fig. 1:**
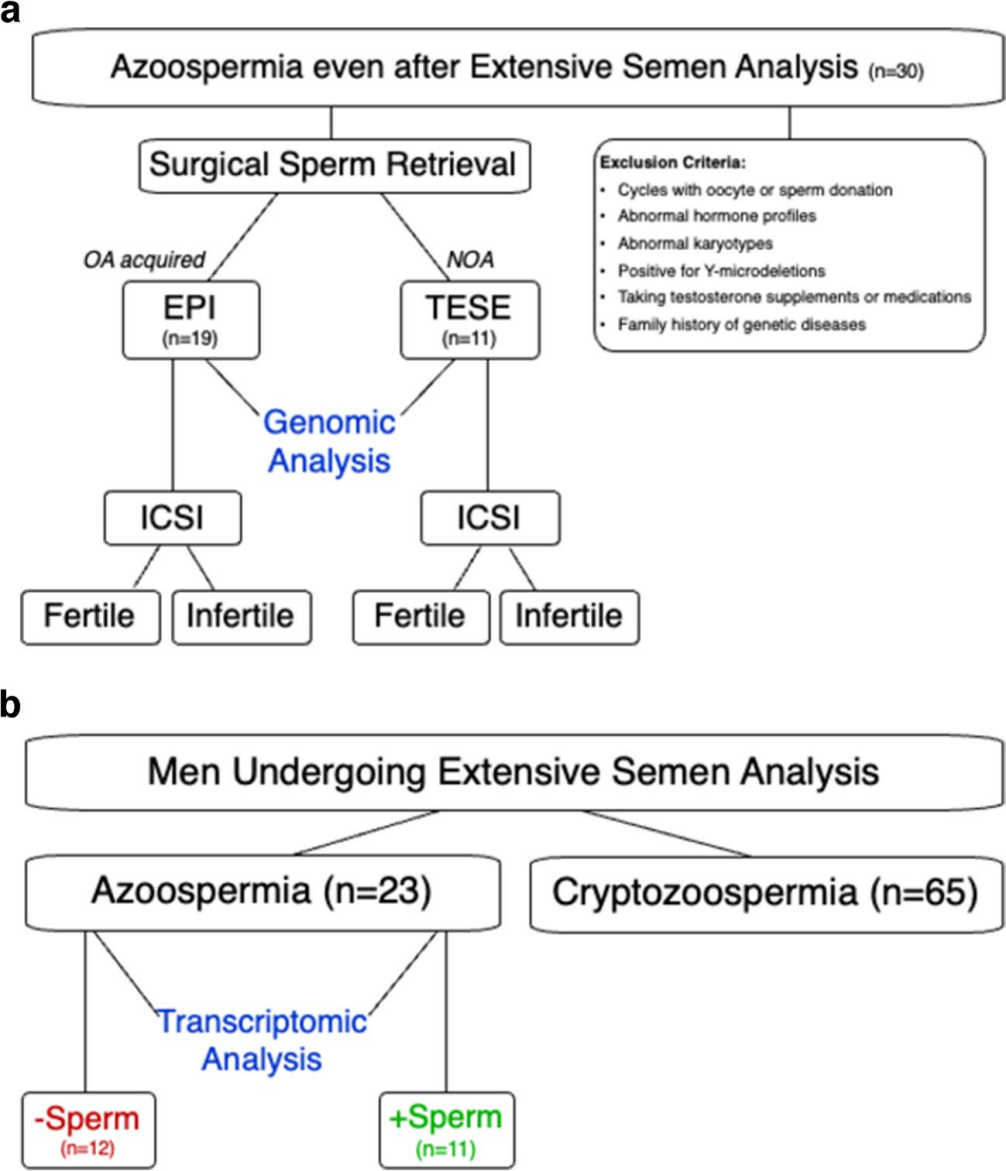
**a** Men undergoing epididymal sperm aspiration for acquired obstructive azoospermia (OA), or testicular biopsy for nonobstructive azoospermia (NOA), were considered eligible for this study. Those for whom spermatozoa were successfully retrieved, and subsequently used for ICSI, were included. Whole exome sequencing (WES) on spermatozoal DNA was performed to compare copy number variants (CNVs) and common germline mutations between the OA and NOA cohorts, as well as according to the couples’ clinical outcomes. **b** RNAseq on seminal fluid from men with azoospermia (*n* = 23) were carried out to compare transcriptomic profiles according to whether spermatozoa were successfully retrieved after testicular biopsy (+ Sperm), or not (− Sperm)

This study was conducted between August 2019 and December 2021 at the Center for Reproductive Medicine of a major academic medical center. Men (37.1 ± 6 years) undergoing epididymal sperm aspiration for obstructive azoospermia (OA; *n* = 19), acquired by vasectomy, or testicular biopsy for nonobstructive azoospermia (NOA; *n* = 11) (36.8 ± 5 years), were considered eligible (Fig. 1a). Those with negative infertility workups, and for whom spermatozoa were successfully retrieved and subsequently used for ICSI, were included. Semen samples from eight fertile normozoospermic men comprised the control group.

Whole exome sequencing (WES) was performed on spermatozoal DNA from surgically retrieved specimens. Copy number variants (CNVs) and common germline mutations were identified and compared between the OA and NOA cohorts, as well as according to the couples’ clinical outcomes, while controlling for maternal age. Those who had successful pregnancies comprised the fertile subgroup, whereas those who were unsuccessful represented the infertile subgroup (Fig. 1a). Although we adopted a complete whole exome sequencing approach, for the purpose of this investigation, we primarily focused on those clinically meaningful genes that were specifically related to spermiogenesis, impaired fertilization, early embryo cleavage, as well as embryo developmental competence.

In a sub-analysis, we included men with suspected azoospermia (*n* = 88), for whom no spermatozoa were seen at initial semen analysis. For some men (*n* = 65), cryptozoospermia was detected after extensive semen analysis. For those with confirmed azoospermia (*n* = 23) in which no spermatozoa were identified despite extensive semen analyses, we performed transcriptomic assessments of cell-free RNAs in their seminal fluid. Four normozoospermic men were included as a control group. To identify genomic biomarkers that can predict testicular sperm retrieval outcome, we then compared the transcriptomic profiles of the 23 azoospermic men in relation to whether spermatozoa were successfully retrieved after testicular biopsy [( +)Sperm] or not [( −)Sperm] (Fig. 1b).

This study (IRB 1006011085) was approved by the Institutional Review Board, and all participants provided written informed consent with guaranteed confidentiality.

### Infertility workup

Female infertility evaluation consisted of a comprehensive review of medical history, targeted physical examination, and tests focusing on ovarian reserve, ovulatory function, tubal patency, and uterine structural abnormalities. All couples underwent hormone profiling. Karyotyping was performed to confirm the absence of genetic alterations. All the couples had normal karyotypes and negative infertility workups, with no family history of genetic diseases. Standard male evaluation for azoospermic men was done according to the AUA/ASRM joint male infertility guidelines [21] with G-band analysis of karyotype and Y microdeletion analysis using multiplex PCR as previously described [22]. All male partners tested negative for Y-microdeletions, and none were taking testosterone supplements or medications to improve sperm parameters before or during the study. None of the patients had family history of infertility. Ejaculates were evaluated according to the 6th edition of the World Health Organization standards [23].

### Extensive sperm search

Ejaculates were provided by masturbation following 1–2 days of sexual abstinence and allowed to liquefy at 37°C for at least 15 min. Initial analysis was conducted on 5 μL of the specimen using the Makler® sperm-counting chamber (Sefi Medical Instruments, Ltd., Haifa, Israel). If no spermatozoa were identified, the specimens were diluted in 3:1 Human Tubal Fluid medium (HTF Medium; Irvine Scientific) supplemented with Human Serum Albumin (HSA-Solution; Vitrolife), centrifuged at 3000 × *g* for 10 min, and reassessed. If spermatozoa were still not present, pellets were placed in 8 μL microdrops under oil in ICSI dishes to be searched under an inverted microscope at 400 × magnification.

### Surgical sperm retrieval

Extensive sperm search was performed to exclude cryptozoospermia. Once azoospermia was confirmed, microdissection testicular sperm extraction (mTESE) was carried out as previously described [24, 25]. All surgeries were performed by a single urologist. Testicular samples were taken from only the most normal appearing tubules, distinguished by their larger size and greater opacity [26]. Tissue was dispersed in 300–400 μL of human tubal fluid medium (Irvine Scientific; Irvine, CA) and mechanically dispersed for several minutes by extensively cutting tissue into small pieces and passing the suspension through a 24G angiocatheter to assure complete disruption of tubules [27]. No other mechanical processing was done. Initial assessment for the presence of spermatozoa was carried out on glass slides using a phase contrast microscope (Olympus BX40) under 200× magnification. If no spermatozoa were identified, further extraction from the same testis and eventually the contralateral testis was performed. When no spermatozoa were identified, the testicular tissue suspension was digested with collagenase for 1 h, during which the suspension was mixed every 10–15 min to enhance enzymatic digestion [28]. An extensive search was subsequently carried out by several well-trained clinical embryologists to identify spermatozoa useful for ICSI.

Epididymal sperm aspiration was performed using a microsurgical technique as previously described [29, 30], where epididymal tubules were dissected and carefully punctured using an ophthalmic microknife (AccuSharp, Redmond, WA). The spermatozoa were then aspirated using micropipettes with 250–350 μm tip widths hand-drawn from glass tubing with an inner diameter of 0.6 mm and outer diameter of 0.9 mm (Drummond Scientific, Broomall, PA) [31]. Further epididymal tubule incisions were made to obtain spermatozoa of optimal quality, indicated by acceptable morphology (no major head defects and full-length sperm tail) and motility adequate for ICSI. Motility was considered present if spermatozoa displayed kinetic characteristics ranging from twitching in place, or progressive according to the intensity of the motion or displacement, since this displays proof of sperm viability [2]. All surgical samples were transported in 0.5 ml of human tubal fluid medium (Irvine Scientific; Irvine, CA) from the operating room to a sterile laboratory, where they were cryopreserved by slowly adding sperm cryopreservation medium (Irvine Scientific; Irvine, CA) until a 1:1 ratio of medium to sperm sample was achieved. After mixing, samples were transferred into labeled cryovials and stored in liquid nitrogen vapor until use in subsequent ICSI cycles.

### Genetic profiling of surgically retrieved spermatozoa

DNA extraction and amplification were carried out using a commercial kit (Repli-G Single Cell; Qiagen, Hilden, Germany) [17], on 500 spermatozoa/specimen individually aspirated with an ICSI pipette. Specimens were sent to an external facility (Genewiz, Inc.; South Plainfield, NJ), where they underwent 150-bp paired-end exome sequencing on an Illumina HiSeq 2500 platform. Reads were trimmed to remove poor-quality nucleotides (error rate < 0.01), and quality assessments of each indexed sample were performed by qPCR (KAPA SYBR Faster Master Mix; Roche, Basel, Switzerland) with two primers (qPCR primer 1.1: AATGATACGGCGACCACCGAGAT, qPCR primer 1.2: CAAGCAGAAGACGGCATACGA). Resulting nM amounts were assessed to confirm successful adapter ligation. A high quality, average coverage of 85 × was obtained for the specimens, with > 90% exome coverage (Agilent SureSelect Human All Exon V6). The base calling accuracy for all samples was ~ 99.9%, as indicated by an average Phred quality score of Q38. After CNV detection was completed using CLC Genomics Server 9.0, the detected variants were annotated to identify gene mutations. All genomic coordinates were based on the human genome assembly GRCh38 (hg38).

### Transcriptomic profiling of seminal plasma from NOA men

Total RNA was isolated and purified from the seminal plasma of men with confirmed azoospermia (*n* = 23) using an RNeasy Mini Kit spin column (RNeasy; Qiagen, Hilden, Germany) as previously described [32]. Nucleic acid quantification was performed with an Agilent 2100 bioanalyzer to determine the RNA integrity number (RIN), while RNA concentration was determined by a NanoDrop spectrophotometer and confirmed by Quibit RNA assay. After library prep (NEBNext Ultra RNA Library Prep kit, New England BioLabs Inc., Ipswich, MA), ribosomal RNAs were isolated by rRNA depletion (Ribo-Zero Gold rRNA Removal kit, Illumina, San Diego, CA). Sequencing was performed (NextSeq500; Illumina, San Diego, CA) by Genewiz, at a pilot-paired end 36 bp before being expanded to 50–60 M reads at 2 × 75 bp. Sequenced reads were then trimmed to remove low quality bases using Trimmomatic v.0.36 and mapped to the hg20 reference genome by CLC Genomics Server 9.0.

### Ovarian superovulation and oocyte collection

Complete descriptions of the stimulation protocol and oocyte collection can be found in previous reports from our institution [33]. Briefly, patients were treated with daily gonadotropins (Follistim, Merck, Kenilworth, NJ, USA; Gonal-F, EMD-Serono, Geneva, Switzerland; and/or Menopur, Ferring Pharmaceuticals Inc, Parsippany, NJ, USA), and pituitary suppression was achieved by GnRH-antagonist (Ganirelix acetate, Merck, Kenilworth, NJ, USA; or Cetrotide, EMD-Serono Inc., Rockland, MA, USA). To attain follicular synchronization, some patients were treated with oral contraceptive pills (Ortho-Novum, Janssen Pharmaceuticals, Beerse, Belgium) prior to starting gonadotropins. The human chorionic gonadotropin trigger (hCG, Ovidreal, EMD Serono) was administered when at least 2 lead follicles reached an average diameter of ≥ 17 mm, and oocyte retrieval was subsequently performed 35–37 h afterwards, transvaginally under conscious sedation. Oocytes were incubated for 3–4 h, then washed in culture medium (home-brew, modified Cornell medium based on G1 and G2 components; Vitrolife, Sweden) [34, 35] and examined under an inverted microscope (TE2000U, Nikon USA, Melville, NY, USA) equipped with 2 × , 4 × , 10 × , 20 × , and 40 × objectives (Nikon CFI Apo & Nikon Polarized optics CFI Plan Fluor). Oocytes with extrusion of the first polar body (PB) were considered to be at the MII stage and ready for ICSI.

### Embryo transfer and assessment of clinical outcome

Successful fertilization was assessed using an inverted microscope (TE2000U, Nikon USA, Melville, NY, USA) equipped with 2 × , 4 × , and 10 × objectives (Nikon CFI Apo), and 20 × and 40 × objectives (Nikon Polarized optics CFI Plan Fluor) [36]. In preparation for embryo transfer, patients received 50 mg of intramuscular progesterone supplement daily, starting from the day after ovulation. The overwhelming majority of patients (*n* = 26) underwent single embryo transfers of blastocysts on day 5, while the remaining (*n* = 4) transferred 2 embryos at the cleavage stage, on day 3. None of the couples included in this study opted for PGT-A testing. Serum βhCG levels were measured between 10 and 14 days post-embryo transfer. Clinical pregnancy was defined as fetal heart activity (+ FHB) detected on ultrasound at 7 weeks of gestation.

### Bioinformatics and statistical analysis

CNV calling and gene mutation annotation was carried out using CLC Genomics Server 9.0 modules including NGS core tools/mapping and re-sequence analysis. Sperm aneuploidy was assessed by calculating the proportion of chromosomal abnormalities detected by CNV analysis [37]. The CNVs were then ranked according to these log-ratio values and corresponding genes annotated. Statistical thresholds of *P* < 0.0005 for significance and *Q* < 0.05 for false positive discovery were used. Sperm genetic profiles were compiled by identifying the mutations that were commonly carried by the spermatozoa from all men within the same group or subgroup.

For differential expression analysis, raw read counts were uploaded according to the DESeq2 v1.23.1 (LGPL, Bioconductor) pipeline and calculated in fragments per kilobase of exon of transcript per million mapped reads (FPKM). After data normalization, gene expression comparison was performed. To avoid over- or under-representing FPKM, an algorithm by edgeR (LGPL; Bioconductor) and CONTRA was implemented following the DESeq2 expression analysis to overcome experimental conditions such as fragmentation [32]. Power analyses were performed using STATA (Stata/BE 17; StataCorp LP, College Station, TX). The Mann–Whitney *U* test and two-tailed *t* test were used to compare the sperm aneuploidy between the NOA and OA cohorts (Graphpad Software, San Diego, CA). A *P* value of < 0.05 was considered statistically significant.

## Results

A total of 30 men were enrolled in this study. Of these, 19 underwent epididymal sperm retrieval for acquired obstructive azoospermia (OA) due to vasectomy, yielding a sperm concentration of 1.1 ± 4 × 10^6^/mL with 9 ± 12% motility. The remaining 11 men underwent testicular biopsies for nonobstructive azoospermia (NOA), yielding a sperm concentration of 0.03 ± 0.4 × 10^6^/mL and 0.5 ± 1% motility (Table 1).

**Table 1:** Patient demographics and surgical sperm parameters

Couples	30	
Maternal age (M years ± SD)	37.8 ± 5	
Paternal age (M years ± SD)	38.4 ± 6	
Surgical sperm retrieval	Obstructive	Non-obstructive
Patients	19	11
Concentration (M × 10^6^/ml ± SD)	1.1 ± 4	0.03 ± 0.4
Motility (M% ± SD)	9.0 ± 12	0.5 ± 1

Out of 30 men, 19 underwent epididymal sperm retrievals, yielding a sperm concentration of 1.1 ± 4 × 10^6^/ml with 9.0 ± 12% motility. The remaining 11 underwent testicular biopsies, yielding a sperm concentration of 0.03 ± 0.4 × 10^6^/ml and 0.5 ± 1% motility

Spermatozoal DNA extraction and amplification from surgically retrieved specimens resulted in an average DNA concentration of 742 ± 520 ng/μL of good quality, confirmed by a 260/280 absorbance ratio of > 1.7. Copy number variant (CNV) assessment using WES did not indicate any significant differences in sperm aneuploidy between the two etiologies (OA, 1.7%; NOA, 1.8%) and control (1.1%).

**Fig. 2:**
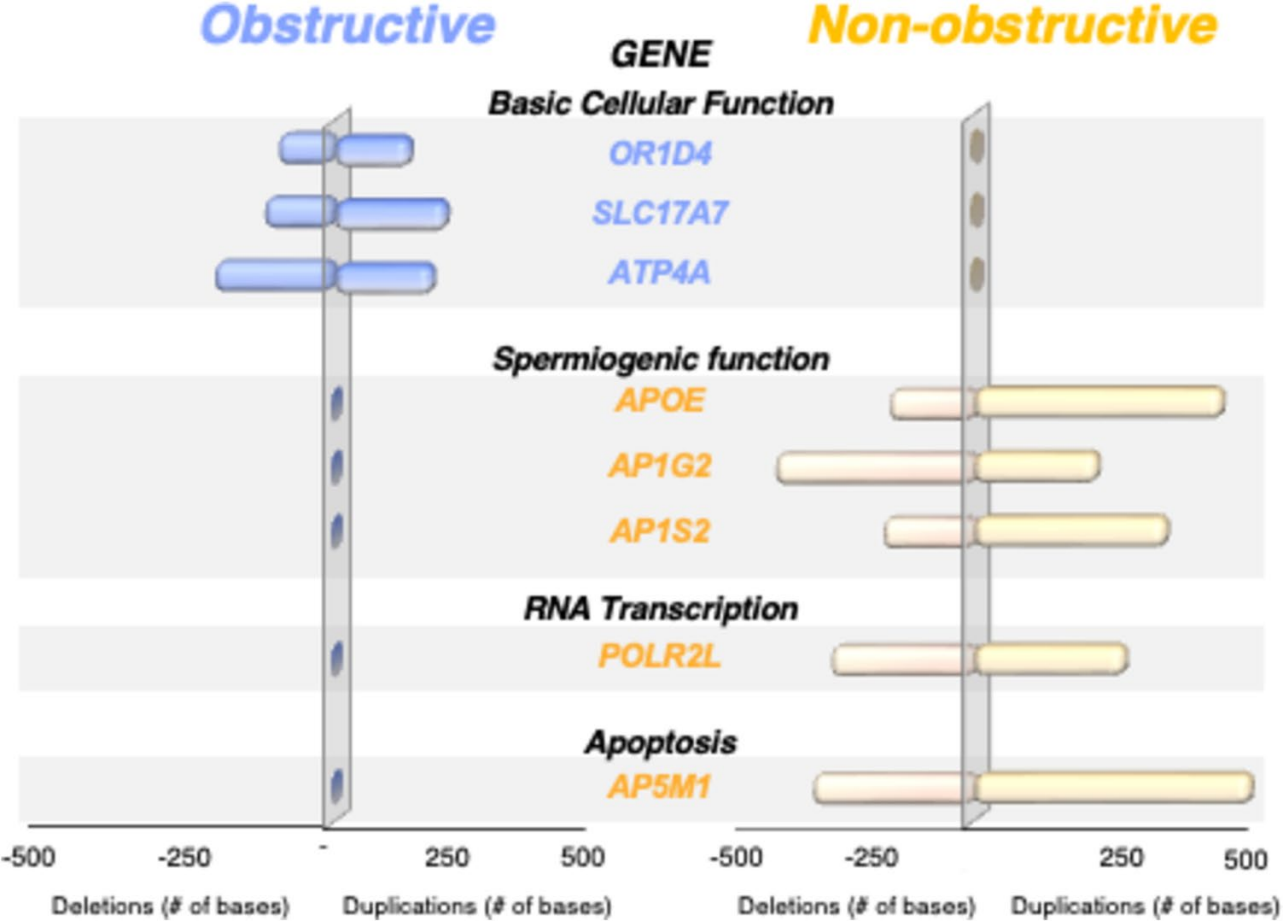
Gene duplications and deletions were compared according to azoospermia etiology. Spermatozoa from the obstructive azoospermia (OA) cohort exclusively carried mutations on 3 housekeeping-related genes (*OR1D4*, *SLC17A7*, *ATP4A*) that are unrelated to sperm production or reproductive potential. Contrastingly, spermatozoa from all men in the nonobstructive azoospermia (NOA) group displayed mutations on 5 genes involved in RNA transcription (*POLR2L*), apoptosis (*AP5M1*), and crucial spermiogenic functions (*AP1S2*, *AP1G2*, *APOE*). These mutations were not identified in specimens from the OA cohort

We then analyzed the annotated CNVs to compare the genetic profiles of the spermatozoa according to the etiology of azoospermia. Overall, our germline mutation analysis of spermatozoa from men in the OA cohort identified an average of 261.8 ± 297 gene mutations/patient, including 19.5 ± 3 that were clinically significant. Of these, we identified three genes (*OR1D4*, *SLC17A7*, and *ATP4A*) that were concurrently mutated in all men with OA. These genes were involved in basic cellular processes and were unrelated to sperm production or reproductive potential. Conversely, spermatozoa from men in the NOA cohort carried a higher occurrence of mutations (884.4 ± 103) (*P* < 0.05) than their OA counterparts, of which 7.1% were clinically meaningful (62.5 ± 21) (*P* < 0.0001). Moreover, across the entire NOA cohort, frameshift mutations were identified in five genes crucial for spermiogenic function (*AP1S2*, *AP1G2*, *APOE*), RNA transcription (*POLR2L*), and apoptosis (*AP5M1*) (Fig. 2 and Table 2).

**Table 2:** Gene mutations according to azoospermia etiology

Obstructive			
Gene	Chr	Location	Description
*OR1D4*	17	n.-65_*298dup	Odorant receptor
*SLC17A7*	19	c.1470 T > G	Mediates uptake of glutamate to synaptic vesicles of neural cells
*ATP4A*	19	c.2006 + 12_2006 + 13insC	Catalyzes ATP hydrolysis with exchange of H( +) and K( +) ions across plasma membrane

Nonobstructive			
Gene	Chr	Location	Description
*APOE*	19	c.43 + 25_43 + 26insC	Spermiogenic function
*AP1G2*	14	c.1345_1346insG	Spermiogenic function
*AP1S2*	X	c.180-22_180-21insT	Spermiogenic function
*AP5M1*	14	c.663A > G	Apoptosis
*POLR2L*	11	c.96-99delG	RNA transcription

Spermatozoa from the obstructive cohort carried mutations on housekeeping genes that were unrelated to spermatogenesis or reproductive function. Spermatozoa from the nonobstructive cohort exclusively displayed mutations on genes involved in RNA transcription, apoptosis, and crucial spermiogenic functions

**Fig. 3:**
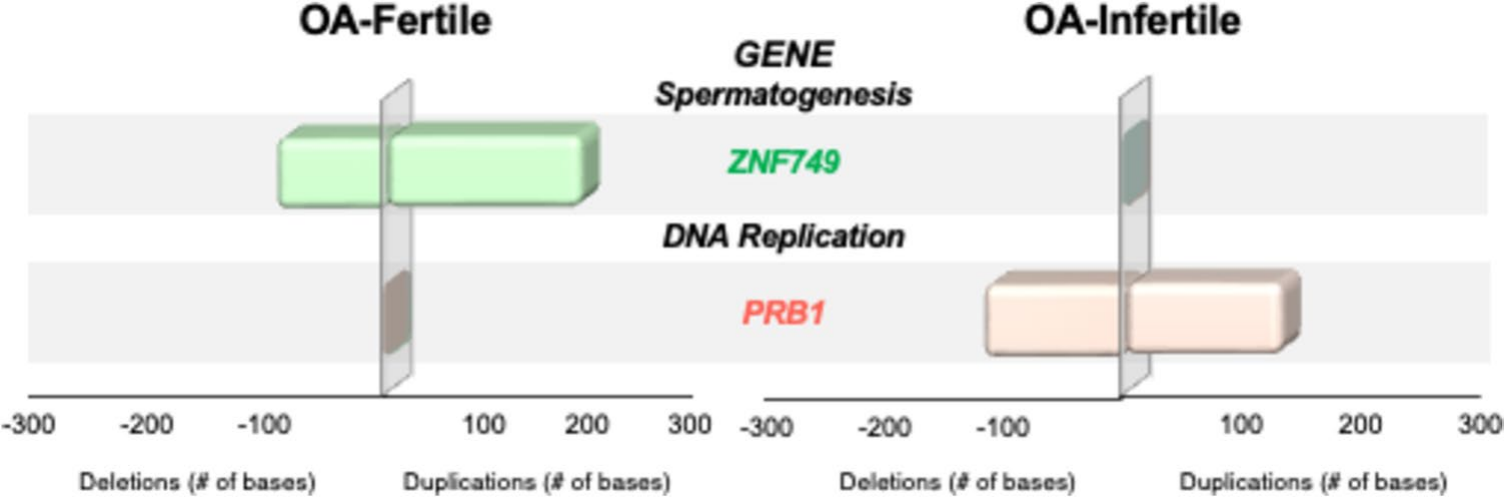
Spermatozoa from the fertile obstructive azoospermia (OA-fertile) subgroup carried mutations on a spermatogenesis-related gene (*ZNF749*), while their infertile obstructive azoospermia (OA-infertile) counterparts displayed mutations on a gene (*PRB1*) responsible for controlling essential DNA replication

To further investigate the genetic causes that may impair the reproductive potential of these surgically retrieved spermatozoa, we categorized couples from the OA and NOA cohorts into subgroups, according to their reproductive outcomes (Table 3). Couples from the OA cohort (maternal age, 36.5 ± 3 years; paternal age, 36.8 ± 7 years) each underwent one ICSI cycle, resulting in a delivery rate of 47.4% (9/19). Spermatozoa isolated from the OA-fertile subgroup (*n* = 9) carried synonymous mutations in a gene specifically affecting sperm production (*ZNF749*), which was not identified in any other subgroup. Spermatozoa from OA-infertile individuals (*n* = 10) displayed missense mutations in a gene responsible for controlling essential DNA replication (*PRB1*), which was also not identified in any other subgroup (Fig. 3).

**Table 3:** ICSI outcomes of study cohorts

	Obstructive		Non-obstructive	
	Fertile	Infertile	Fertile	Infertile
Couples	9	10	8	3
Maternal age (M ± SD)	35.8 ± 4	36.5 ± 3	36.8 ± 2	36.7 ± 1
Paternal age (M ± SD)	36.8 ± 7	37.5 ± 6	37.1 ± 6	36.3 ± 5
Cycles	9	10	8	3
Oocytes retrieved	129	96	147	46
Fertilization (%)	94/117 (80.3)	52/75 (69.3)	81/121 (66.9)	22/41 (53.7)
Cycles with ET	9	10	8	3
Clinical pregnancy (+ FHB) (%)	9 (100)	0	8 (100)	0
Deliveries	9	-	8	-

To further investigate the genetic causes that may impair the reproductive potential of these surgically retrieved spermatozoa, couples from the Obstructive Azoospermia and Nonobstructive Azoospermia cohorts were divided into subgroups according to their ICSI clinical outcomes. Those who generated successful pregnancies comprised the fertile subgroup, while those who were unsuccessful represented the infertile subgroup

**Fig. 4:**
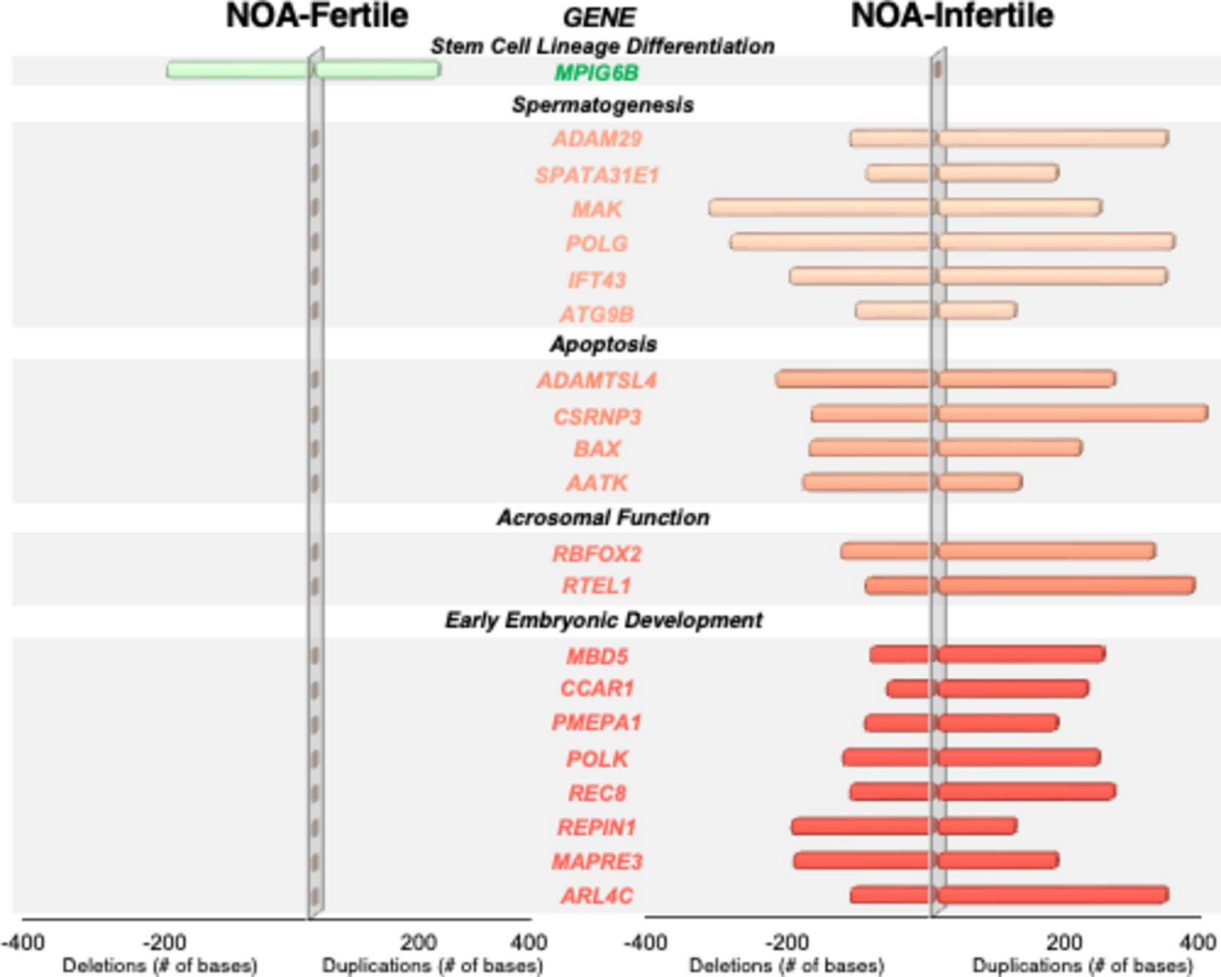
Spermatozoa from the fertile nonobstructive azoospermia (NOA-fertile) subgroup carried mutations on a single gene (*MPIG6B*) involved in stem cell lineage differentiation, while spermatozoa from each of the infertile counterparts displayed mutations on genes not only involved in spermatogenesis, but also apoptosis, acrosomal function, and most importantly, early embryonic development

Couples from the NOA cohort (maternal age, 36.8 ± 2 years; paternal age, 37.1 ± 6) underwent 11 ICSI cycles, yielding a delivery rate of 72.7% (8/11) (Table 3). Spermatozoa from the NOA-fertile men (*n* = 8) all carried mild mutations on *MPIG6B*, a gene involved in stem cell lineage differentiation, while gametes from each of their NOA-infertile counterparts (*n* = 3) displayed frameshift and point mutations on genes involved in spermato-/spermiogenesis (*ADAM29*, *SPATA31E1*, *MAK*, *POLG*, *IFT43*, *ATG9B*), apoptosis (*ADAMTSL4*, *CSRNP3*, *BAX*, *AATK*), and acrosomal function (*RBFOX2*, *RTEL1*). Most importantly, they carried severe mutations in the genes that encode early embryonic development (*MBD5*, *CCAR1*, *PMEPA1*, *POLK*, *REC8*, *REPIN1*, *MAPRE3*, *ARL4C*) (Fig. 4).

Next, we performed a sub-analysis using RNAseq to determine whether the transcriptomic profiles of seminal plasma from men can predict successful sperm extraction at testicular biopsy. For this assessment, we included a total of 88 men (37.3 ± 6 years) with suspected azoospermia. We performed extensive semen analyses of their ejaculates and identified rare spermatozoa in the majority (*n* = 65) of these patients, whereas spermatozoa were not identified in the remaining 23 men. Therefore, these 23 patients with confirmed azoospermia subsequently underwent testicular biopsy.

RNAseq was performed on circulating cell-free RNA in the seminal fluid from each azoospermic patient (concentration 9.1 ± 4 ng/μL). Eleven men (39.0 ± 12 years) who underwent successful testicular sperm retrievals were defined as the ( +)Sperm cohort, while 12 (34.3 ± 5 years) with unsuccessful outcomes comprised the ( −)Sperm cohort. Transcriptomic analyses of seminal plasma from the ( +)Sperm cohort identified 12 significantly imbalanced genes that were exclusively shared by all men in this group. These genes were primarily involved in spermatogenesis (*PRM2*, *KLHL10*, *SPZ1*, *SPATA12*, *H1FNT*), sperm maturation (*ADAM21*), cell cycle regulation (*CENPU*, *TSPAN6*, *BOD1L2*), and sperm function (*TTC29*, *ZBBX*, *WBP2NL*) (Table 4).

**Table 4:** Significant differentially expressed genes in nonobstructive azoospermic (NOA) men with successful TESE outcomes compared to a normozoospermic control

Gene	Chr	Log2 Fold Change	Description
*PRM2*	16	− 10.2	Substitute histones in sperm chromatin during haploid phase of spermatogenesis
*KLHL10*	17	− 4.2	Mediates proteasomal degradation during spermatogenesis
*SPZ1*	5	− 4.1	Regulation of cell proliferation and differentiation during spermatogenesis
*SPATA12*	3	− 5.1	Highly expressed in testis; testicular development, spermatogenesis
*H1FNT*	12	− 5.4	DNA condensation during spermatogenesis
*ADAM21*	14	− 3.8	Implicated in sperm maturation and fertilization
*CENPU*	4	− 1.2	Involved in cell cycle
*TSPAN6*	X	2.2	Cell development, cell growth
*BOD1L2*	18	− 8.3	Chromosome bio-orientation
*TTC29*	4	− 1.3	Implicated in sperm motility
*ZBBX*	3	3.1	Involved in cilium movement
*WBP2NL*	22	− 4.4	Meiotic resumption during fertilization; sperm specific

Seminal plasma obtained from the + TESE patients exclusively shared 12 significantly imbalanced genes that are mainly involved in spermatogenesis, sperm maturation, and cell cycle regulation

Transcriptomic analyses of the (-)Sperm cohort identified 19 significantly imbalanced genes that were exclusively shared in the seminal plasma. These genes were mainly involved in spermatogenesis (*PHF7*, *GAPDHS*, *ACSBG2*, *CABS1*, *TPD52L3*, *PGK2*, *CAPZA2*, *TSSK6*, *UBQLN3*, *TEX44*, *SPATA42*), sperm function (*TCP11*, *CABYR*, *ODF1*, *ODF3L2*, *FSCB*, *AKAP4*, *TPPP2*), and testis development (*SPEM2*) (Table 5).

**Table 5:** Significant differentially expressed genes in nonobstructive azoospermic (NOA) men with unsuccessful TESE outcomes compared to a normozoospermic control

Gene	Chr	Log2 Fold Change	Description
*PHF7*	3	− 1.7	Spermatogenesis, expressed in Sertoli cells
*GAPDHS*	19	− 4.6	Implicated in energy production for spermiogenesis
*ACSBG2*	19	− 5.7	Implicated in spermatogenesis
*CABS1*	4	− 6.2	Involved in spermatogenesis
*TPD52L3*	9	− 8.2	May play a role in spermatogenesis
*PGK2*	6	− 7.0	Specific expression in the testis; spermatogenesis
*CAPZA2*	12	− 0.1	Encodes actin capping protein; sperm architecture
*TSSK6*	19	− 6.5	Role in sperm production and DNA condensation
*UBQLN3*	11	− 4.1	Cell cycle progression during spermatogenesis, specific expression in testis
*TEX44*	2	− 9.2	Strong expression in elongating spermatids
*SPATA42*	1	− 9.9	Associated with Spermatogenic Failure
*TCP11*	6	− 4.7	Role in sperm capacitation and acrosome reaction
*CABYR*	18	− 3.9	Associated with capacitation and acrosome reaction
*ODF1*	8	− 7.4	Sperm motility
*ODF3L2*	19	− 7.9	Outer dense fiber of sperm tails
*FSCB*	14	− 9.0	Fibrous sheath biogenesis and capacitation
*AKAP4*	X	− 10.1	Sperm motility
*TPPP2*	14	− 4.6	Sperm motility
*SPEM2*	17	− 7.7	Highly expressed in testis

For the men who underwent unsuccessful testicular sperm retrievals, we identified 19 significantly imbalanced genes that were exclusively shared in their seminal plasma. These genes are mainly involved in processes such as spermatogenesis, sperm function, and testis development

**Fig. 5:**
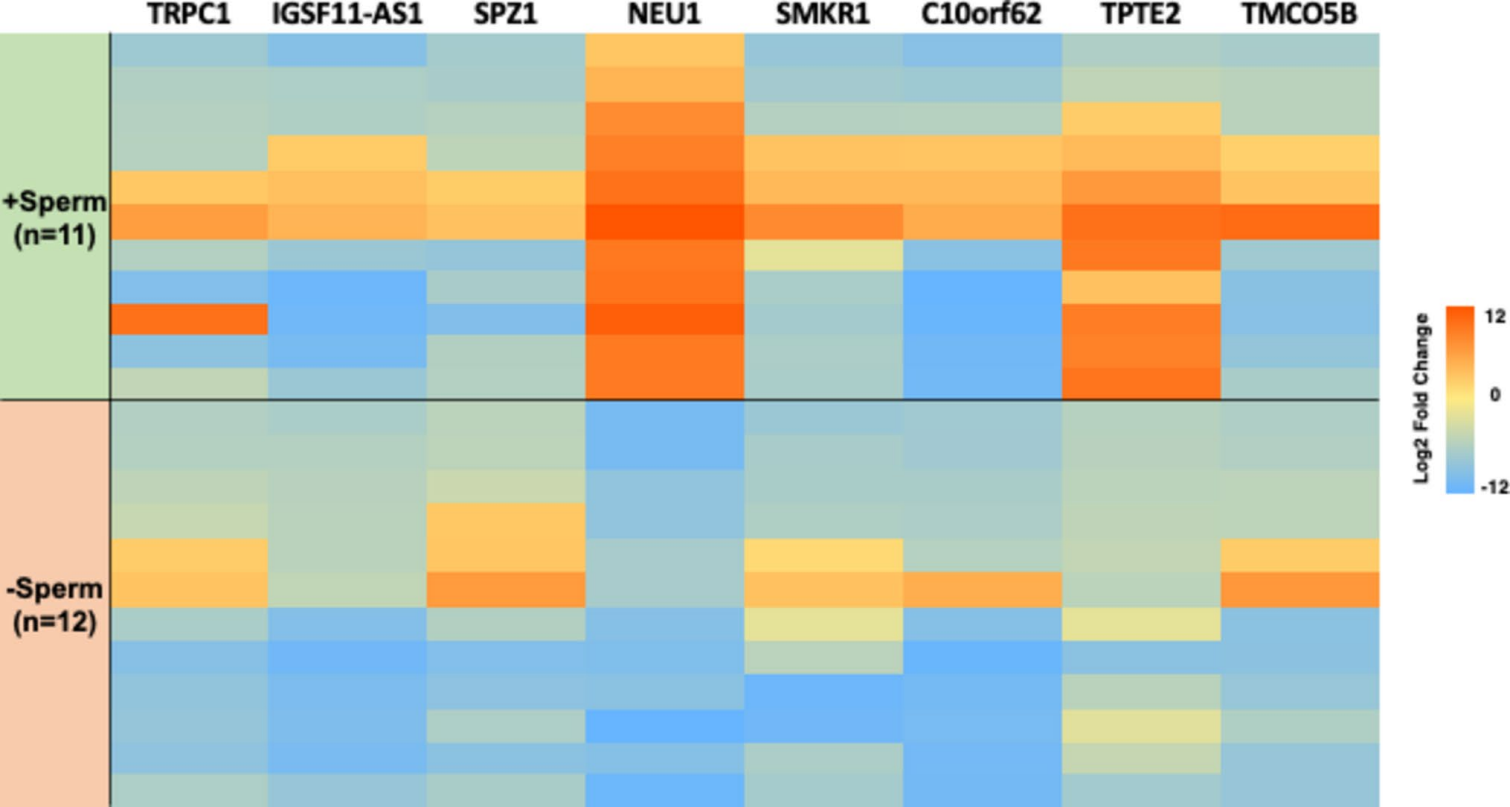
RNAseq was performed on the seminal fluid from 23 azoospermic patients. Eleven men who underwent successful testicular sperm retrievals were defined as the ( +)Sperm cohort, while 12 with unsuccessful outcomes comprised the ( −)Sperm cohort. We identified 8 imbalanced genes that were shared among all azoospermic men. *TPTE2* was partially expressed in the seminal plasma from 81.8% (9/11) of the ( +)Sperm group, while *IGSF11-AS1* was underexpressed in the seminal plasma from all ( −)Sperm men. Most interestingly, *NEU1* was identified as the sole gene that was exclusively underexpressed in all men from the ( −)Sperm cohort, yet simultaneously overexpressed in all men from the ( +)Sperm cohort

Upon comparing the transcriptomic profiles between the ( +) and ( −)Sperm cohorts, we identified eight genes that were significantly over- or underexpressed and shared among the entire azoospermic group (Table 6). These genes are involved in spermatogenesis (*TMCO5B*, *C10orf62*, *SMKR1*, *SPZ1*), sperm function (*NEU1*, *TPTE2*), and testis development (*TRPC1*, *IGSF11-AS1*). A comparison of the expression of each gene between the two subgroups showed that *TPTE2*, implicated in spermatogenic defects and normally highly expressed in the testis, was expressed in the seminal plasma from 81.8% (9/11) of the ( +)Sperm group; most importantly, *NEU1*, involved in acrosome development and crucial for sperm capacitation, was overexpressed in all men from the ( +)Sperm cohort. Interestingly, these two genes, together with *IGSF11-AS1*, were consistently underexpressed in the ( −)Sperm cohort (*P* < 0.0001) (Fig. 5).

**Table 6:** Comparison of significant differentially expressed genes in nonobstructive azoospermic (NOA) men according to TESE outcome

		-Sperm	+ Sperm	
Gene	Chr	Log2 fold Change		Description
*NEU1*	6	− 6.36	9.67	Acrosomal reaction and capacitation
*TPTE2*	13	− 4.01	5.88	Acts as a lipid phosphatase, sperm motility
*TMCO5B*	15	− 5.38	− 3.96	Spermatid development in mouse
*IGSF11-AS1*	3	− 8.79	− 6.04	Long non-coding RNA, downregulated in infertile male
*C10orf62*	10	− 9.02	− 6.81	Spermatid development, testis-specific
*SMKR1*	7	− 5.65	− 2.79	Spermatid development, testis-specific in ovine species
*TRPC1*	3	− 5.37	− 3.16	Transient receptor potential non voltage-channel 1, expressed in adult testis, ovaries
*SPZ1*	5	− 3.93	− 5.03	Regulation of cell proliferation/differentiation during spermatogenesis

Comparison of transcriptomic profiles between the ( +) and ( −)Sperm cohorts identified eight imbalanced genes that were shared among the entire azoospermic group

## Discussion

Despite the evolution of genetic testing in the field of male infertility, some gaps remain in its application, particularly in cases requiring testicular sperm extraction. For instance, the assessment of men with azoospermia is primarily focused on identifying the etiology of compromised sperm production and does not extend to the evaluation of the reproductive potential of surgically retrieved gametes. Earlier studies have assessed the chromosomal profile of testicular spermatozoa [14, 15, 38], but these were carried out using early cytogenetic techniques, yielding inaccurate results that fogged the situation and prompted concerns related to the safety of using such specimens. However, recent evidence has mitigated these concerns by showing that clinical outcomes are not impaired in cycles using testicular spermatozoa compared with those using ejaculated specimens [17].

Recent studies on the genetics of azoospermia have identified specific genes associated with a large spectrum of testis phenotypes, from hypospermatogenesis (*USP9Y*) to residual spermatogenesis (*PRY*, *RBBMY*, *BPY2*, *DAZ*) [39–41]. Rare variants of *ADGRG2* have also been associated with unexplained CBAVD cases that do not display any pathogenic *CFTR* mutations [39]. However, these assessments were primarily performed on somatic tissues. Notably, there is a clear dichotomy between somatic cells and the germline, including the response to environmental stimuli, as well as the type and incidence of mutations associated with the transgenerational role of the latter [42]. Germline and somatic mutations also occur in different settings. Although public databases of somatic and germline mutations indicate many shared variants, this may primarily be attributed to basic chemical vulnerabilities that are common in DNA from both environments [43]. In the current study, we specifically screened spermatozoal DNA to identify germline mutations related to whether azoospermia was due to testicular or post-testicular causes, and to identify key gene–related mechanistic links to the reproductive performance of the surgically retrieved specimens.

Our gamete genome comparison between the two azoospermia etiologies revealed a significantly lower incidence of mutations in the OA cohort than in the NOA cohort. Additionally, the OA cohort concurrently carried mutations in three housekeeping genes (*OR1D4*, *SLC17A7*, *ATP4A*) unrelated to reproductive competence, whereas the NOA cohort displayed frameshift mutations in genes crucial for spermatogenic function (*AP1S2*, *AP1G2*, *APOE*), RNA transcription (*POLR2L*), and apoptosis (*AP5M1*).

We then divided the OA cohort according to ICSI outcomes and found that spermatozoa from all men in the OA-fertile subgroup displayed mutations in a gene (*ZNF749*) from the zinc finger protein family, which has been shown to affect sperm production [44]. This finding was unexpected, given that the OA participants were all post-vasectomies with a history of normal spermatogenesis. However, the mutations were synonymous and would not have disturbed the resulting protein structure. The unaffected protein function, characterized by stable zinc ion activity, also suggests that the subsequent sperm-specific zinc signature crucial for capacitation and sperm-zona pellucida interactions [45] is sustained, which explains why the clinical outcomes were not impaired in this subgroup.

Spermatozoa from all men in the OA-infertile subgroup exclusively carried missense mutations on *PRB1*. While this gene is known for its critical role in clipping of the histone H3 N-terminal tails, thereby maintaining cellular resistance to DNA-damaging agents in *Saccharomyces cerevisiae* [46], its capacity to do so in humans is unknown. However, sperm chromatin fragmentation (SCF) assessment by the TUNEL assay showed elevated SCF, suggesting that *PRB1* alterations carried by spermatozoa from infertile men with obstructive azoospermia, acquired by vasectomy, may affect their ability to withstand the action of oxygen free radicals, subsequently impairing embryo development and precluding these patients from achieving pregnancy [47].

Our sperm genetic assessment for the NOA-fertile subgroup identified mutations in a single gene, *MPIG6B*. Although this gene is primarily involved in hematopoietic lineage differentiation, gene expression clustering has shown that it behaves in a coordinated manner with C*ATSPERD* [48], which is crucial for sperm capacitation and successful fertilization. However, this subgroup obtained a fertilization rate of 66.9%, which suggests that any sperm acrosomal flaws from mutations in *MPIG6B* or other genes within the same cluster were successfully addressed by ICSI, which is known for its ability to overcome defects in sperm-egg fusion and acrosomal development [49]. Indeed, while the NOA-infertile subgroup carried mutations in genes involved in spermato/spermio-genesis (*ADAM29*, *SPATA31E1*, *MAK*, *POLG*, *IFT43*, *ATG9B*), they also displayed additional frameshift and point mutations in genes that encode early embryonic development (*MBD5*, *CCAR1*, *PMEPA1*, *POLK*, *REC8*, *REPIN1*, *MAPRE3*, *ARL4C*), which may be a concurrent compounding factor in the implantation failure observed in these couples.

Next, we investigated whether the gene expression profiling of cell-free RNAs in seminal plasma could be used to predict successful testicular sperm retrieval in men with non-obstructive azoospermia. Although the chance of successful micro-TESE in NOA men can reach 60%, the procedure may still fail to yield spermatozoa. The evaluation of several factors has been proposed to predict successful retrieval, including FSH [50], inhibin B [51], genetics [52], and histopathology [53]. Although histopathology is considered the most reliable method for predicting a successful micro-TESE, it is equally invasive. Our RNAseq assessment of circulating cell-free RNA in seminal plasma identified several differentially expressed genes (DEGs), including those involved in spermatogenesis and sperm function, regardless of whether spermatozoa were successfully retrieved after testicular biopsy. However, the differential expression of a select number of genes distinguished those NOA men who had successful micro-TESE sperm retrievals from those who were unsuccessful. Specifically, genes crucial for sperm maturation and cell cycle regulation were underexpressed in the seminal plasma of men who underwent unsuccessful procedures, suggesting that the probability of retrieving spermatozoa is correlated with the downregulation of genes involved in these functions. This was further illustrated by the expression profile of *NEU1*, which was consistently overexpressed in the seminal plasma from the entire ( +)Sperm cohort, yet jointly underexpressed in all men from the ( −)Sperm cohort. This observation may also warrant further investigation into other genes that are responsible for sperm acrosome function and development.

The limitations of this study stem mainly from the relatively small sample size. Couples were selected according to their willingness to participate, and underwent thorough counseling by a reproductive endocrinologist, reproductive urologist, and research coordinators prior to giving their written consent. There was no difference in demographics between the participants and non-participants. This included ethnicity, socioeconomic status, age, and hormonal profile, AMH level, and BMI of the female partners. Although maternal age was controlled for, confounding factors related to the female partner cannot undoubtedly be excluded. In addition, while we adopted a complete whole exome sequencing approach, for the purpose of this investigation, we primarily focused on those genes suspected to be involved in spermatogenesis and embryo developmental competence. Although spermatozoa from men unable to reproduce displayed common gene mutations that provide information about their condition, these findings should still be prospectively validated. Additionally, somatic DNA analysis could determine whether any of the germline mutations identified in this study overlapped with the somatic mutations of the same genes. A comparison of germline and somatic mutations within the same individual would definitively reveal the relationship between the two types of abnormalities and support the diagnostic value of our germline genomic assessment. It is also important to determine the proportion of gametes that carry these mutations, especially for couples undergoing ICSI, where spermatozoa are individually selected. Therefore, future endeavors would include the utilization of single-cell NGS to explore gamete heterozygosity and mutation rates in individual spermatozoa, due to the inherent variability that has been identified within a spermatozoa population [54]. A concurrent somatic RNAseq analysis could also identify gene imbalances that influence spermatogenesis, which may encourage reconsideration of germline mutations initially dismissed as clinically irrelevant. A confirmatory assessment of circulating cell-free nucleic acids in the seminal fluid of NOA patients may also serve to identify additional predictive biomarkers at the genomic and transcriptional levels. Nevertheless, to our knowledge this is the first study that attempts to attribute reproductive competence to spermatozoa retrieved via surgical intervention by profiling their gene sequences. These findings, combined with our RNAseq observations, can potentially be incorporated into a diagnostic tool to categorize and counsel azoospermic men who are candidates for surgical sperm retrieval. Therefore, screening for DEGs and gene mutations that can predict TESE success and reproductive potential, respectively, can be used as a non-invasive biomarker tool to spare patients from unsuccessful surgery. By querying the sperm genome of surgically retrieved specimens, we identified clear germline mutations related to the origin of azoospermia, and most importantly, the ability to support a healthy pregnancy by pinpointing key gene–related mechanistic links to impaired embryo development. RNAseq of seminal fluid from NOA men also revealed predictive indicators of favorable sperm retrieval at testicular biopsy. In conjunction, these genetic and transcriptomic assessments could potentially serve as a useful precision medicine approach, not only to spare azoospermic men from an unpleasant procedure, but most importantly, to pinpoint the diagnosis and predict clinical outcomes for couples affected by the most severe form of male factor infertility.

### Supplementary Information

Below is the link to the electronic supplementary material.

Supplementary file1 (DOCX 45 kb)
