# Supplementary material for: Genetic profiling of azoospermic men to identify the etiology and predict reproductive potential
Source: J Assist Reprod Genet. 2024 Feb 26;41(4):1111–24. doi: 10.1007/s10815-024-03045-5 (PMC11052749; doi:10.1007/s10815-024-03045-5)
Supplement: Supplementary file 1 — Supplementary file1 (DOCX 45 kb) [file 10815_2024_3045_MOESM1_ESM.docx]

**Supplementary Tables:**

**Supplementary Table 1.** CNVs for Nonobstructive Azoospermic (NOA) cohort

| **ch** | ***NOA1*** | ***NOA2*** | ***NOA3*** | ***NOA4*** | ***NOA5*** | ***NOA6*** | ***NOA7*** | ***NOA8*** | ***NOA9*** | ***NOA10*** | ***NOA11*** |
| --- | --- | --- | --- | --- | --- | --- | --- | --- | --- | --- | --- |
| **1** | 0.03 | 0.02 | 0.02 | 0.01 | 0.13 | 0.02 | 0.01 | 0.01 | 0.01 | 0.01 | 0.01 |
| **2** | 0.06 | 0.00 | 0.04 | 0.02 | 0.07 | 0.02 | 0.01 | 0.01 | 0.01 | 0.01 | 0.00 |
| **3** | 0.00 | 0.01 | 0.01 | 0.01 | 0.13 | 0.14 | 0.11 | 0.08 | 0.07 | 0.07 | 0.03 |
| **4** | 0.01 | 0.04 | 0.02 | 0.03 | 0.09 | 0.07 | 0.03 | 0.08 | 0.06 | 0.05 | 0.01 |
| **5** | 0.07 | 0.22 | 0.09 | 0.02 | 0.17 | 0.00 | 0.19 | 0.09 | 0.07 | 0.00 | 0.05 |
| **6** | 0.03 | 0.01 | 0.01 | 0.01 | 0.15 | 0.14 | 0.11 | 0.10 | 0.07 | 0.08 | 0.00 |
| **7** | 0.41 | 0.05 | 0.41 | 0.48 | 0.00 | 0.12 | 0.15 | 0.10 | 0.08 | 0.08 | 0.06 |
| **8** | 0.12 | 0.05 | 0.13 | 0.02 | 0.15 | 0.19 | 0.13 | 0.10 | 0.07 | 0.08 | 0.05 |
| **9** | 0.16 | 0.00 | 0.12 | 0.00 | 0.10 | 0.11 | 0.07 | 0.00 | 0.09 | 0.08 | 0.05 |
| **10** | 0.10 | 0.04 | 0.04 | 0.04 | 0.14 | 0.04 | 0.12 | 0.00 | 0.09 | 0.08 | 0.06 |
| **11** | 0.06 | 0.00 | 0.03 | 0.00 | 0.46 | 0.22 | 0.21 | 0.12 | 0.10 | 0.06 | 0.08 |
| **12** | 0.08 | 0.01 | 0.04 | 0.02 | 0.19 | 0.17 | 0.19 | 0.11 | 0.09 | 0.09 | 0.04 |
| **13** | 0.00 | 0.03 | 0.00 | 0.00 | 0.10 | 0.10 | 0.00 | 0.09 | 0.06 | 0.05 | 0.02 |
| **14** | 0.14 | 0.01 | 0.11 | 0.02 | 0.10 | 0.07 | 0.06 | 0.11 | 0.00 | 0.05 | 0.00 |
| **15** | 0.14 | 0.03 | 0.15 | 0.01 | 0.09 | 0.00 | 0.19 | 0.10 | 0.00 | 0.09 | 0.10 |
| **16** | 0.08 | 0.01 | 0.08 | 0.00 | 0.11 | 0.21 | 0.13 | 0.12 | 0.11 | 0.06 | 0.11 |
| **17** | 0.06 | 0.02 | 0.02 | 0.00 | 0.08 | 0.08 | 0.12 | 0.11 | 0.10 | 0.04 | 0.11 |
| **18** | 0.00 | 0.00 | 0.00 | 0.00 | 0.14 | 0.27 | 0.14 | 0.10 | 0.09 | 0.00 | 0.07 |
| **19** | 0.13 | 0.01 | 0.03 | 0.01 | 0.08 | 0.04 | 0.09 | 0.14 | 0.13 | 0.02 | 0.11 |
| **20** | 0.06 | 0.01 | 0.03 | 0.00 | 0.08 | 0.18 | 0.16 | 0.10 | 0.10 | 0.11 | 0.11 |
| **21** | 0.00 | 0.04 | 0.00 | 0.02 | 0.11 | 0.26 | 0.24 | 0.11 | 0.12 | 0.11 | 0.08 |
| **22** | 0.18 | 0.04 | 0.11 | 0.01 | 0.08 | 0.15 | 0.12 | 0.11 | 0.11 | 0.05 | 0.11 |
| **X** | 0.32 | 0.08 | 0.00 | 0.05 | 0.05 | 0.07 | 0.00 | 0.00 | 0.05 | 0.00 | 0.03 |
| **Y** | 0.29 | 0.00 | 0.18 | 0.16 | 0.00 | 0.00 | 0.00 | 0.00 | 0.00 | 0.14 | 0.00 |

**Supplementary Table 2.** CNV values for Obstructive Azoospermic (OA) cohort

| **ch** | ***OA1*** | ***OA2*** | ***OA3*** | ***OA4*** | ***OA5*** | ***OA6*** | ***OA7*** | ***OA8*** | ***OA9*** | ***OA10*** | ***OA11*** | ***OA12*** | ***OA13*** | ***OA14*** | ***OA15*** | ***OA16*** | ***OA17*** | ***OA18*** | ***OA19*** |
| --- | --- | --- | --- | --- | --- | --- | --- | --- | --- | --- | --- | --- | --- | --- | --- | --- | --- | --- | --- |
| **1** | 0.01 | 0.03 | 0.03 | 0.02 | 0.06 | 0.03 | 0.02 | 0.03 | 0.07 | 0.05 | 0.07 | 0.09 | 0.01 | 0.01 | 0.05 | 0.08 | 0.01 | 0.01 | 0.07 |
| **2** | 0.02 | 0.01 | 0.06 | 0.04 | 0.05 | 0.09 | 0.00 | 0.06 | 0.03 | 0.01 | 0.10 | 0.11 | 0.01 | 0.01 | 0.05 | 0.08 | 0.01 | 0.01 | 0.05 |
| **3** | 0.33 | 0.01 | 0.00 | 0.01 | 0.33 | 0.02 | 0.01 | 0.00 | 0.03 | 0.01 | 0.02 | 0.12 | 0.07 | 0.07 | 0.07 | 0.09 | 0.11 | 0.08 | 0.06 |
| **4** | 0.04 | 0.03 | 0.09 | 0.02 | 0.04 | 0.03 | 0.01 | 0.01 | 0.02 | 0.00 | 0.06 | 0.07 | 0.04 | 0.07 | 0.08 | 0.10 | 0.03 | 0.07 | 0.05 |
| **5** | 0.04 | 0.01 | 0.07 | 0.09 | 0.04 | 0.01 | 0.22 | 0.01 | 0.04 | 0.04 | 0.09 | 0.08 | 0.06 | 0.08 | 0.01 | 0.08 | 0.06 | 0.08 | 0.06 |
| **6** | 0.10 | 0.03 | 0.09 | 0.09 | 0.10 | 0.03 | 0.01 | 0.03 | 0.09 | 0.03 | 0.06 | 0.01 | 0.09 | 0.09 | 0.09 | 0.09 | 0.10 | 0.08 | 0.06 |
| **7** | 0.02 | 0.12 | 0.02 | 0.41 | 0.02 | 0.12 | 0.05 | 0.41 | 0.05 | 0.09 | 0.10 | 0.13 | 0.23 | 0.08 | 0.09 | 0.10 | 0.16 | 0.09 | 0.07 |
| **8** | 0.03 | 0.04 | 0.12 | 0.13 | 0.03 | 0.04 | 0.05 | 0.12 | 0.38 | 0.19 | 0.01 | 0.10 | 0.09 | 0.07 | 0.08 | 0.10 | 0.06 | 0.08 | 0.01 |
| **9** | 0.07 | 0.04 | 0.15 | 0.01 | 0.02 | 0.04 | 0.00 | 0.15 | 0.33 | 0.09 | 0.05 | 0.05 | 0.01 | 0.09 | 0.09 | 0.09 | 0.10 | 0.10 | 0.07 |
| **10** | 0.04 | 0.04 | 0.01 | 0.04 | 0.04 | 0.04 | 0.04 | 0.08 | 0.02 | 0.11 | 0.10 | 0.02 | 0.02 | 0.08 | 0.01 | 0.10 | 0.01 | 0.09 | 0.07 |
| **11** | 0.06 | 0.03 | 0.02 | 0.03 | 0.06 | 0.03 | 0.00 | 0.05 | 0.04 | 0.01 | 0.20 | 0.21 | 0.15 | 0.10 | 0.01 | 0.01 | 0.20 | 0.10 | 0.08 |
| **12** | 0.13 | 0.08 | 0.08 | 0.04 | 0.13 | 0.08 | 0.01 | 0.08 | 0.04 | 0.21 | 0.06 | 0.09 | 0.13 | 0.12 | 0.09 | 0.10 | 0.21 | 0.10 | 0.01 |
| **13** | 0.01 | 0.00 | 0.00 | 0.00 | 0.01 | 0.00 | 0.03 | 0.00 | 0.03 | 0.09 | 0.05 | 0.06 | 0.07 | 0.11 | 0.08 | 0.11 | 0.05 | 0.08 | 0.02 |
| **14** | 0.15 | 0.05 | 0.02 | 0.11 | 0.15 | 0.05 | 0.01 | 0.14 | 0.12 | 0.06 | 0.01 | 0.16 | 0.01 | 0.09 | 0.10 | 0.09 | 0.17 | 0.10 | 0.07 |
| **15** | 0.04 | 0.03 | 0.14 | 0.15 | 0.04 | 0.03 | 0.03 | 0.14 | 0.10 | 0.06 | 0.01 | 0.03 | 0.08 | 0.11 | 0.01 | 0.09 | 0.25 | 0.10 | 0.02 |
| **16** | 0.02 | 0.01 | 0.02 | 0.08 | 0.02 | 0.13 | 0.01 | 0.08 | 0.06 | 0.09 | 0.06 | 0.09 | 0.18 | 0.09 | 0.08 | 0.08 | 0.15 | 0.11 | 0.09 |
| **17** | 0.02 | 0.03 | 0.06 | 0.02 | 0.12 | 0.03 | 0.02 | 0.06 | 0.16 | 0.00 | 0.06 | 0.08 | 0.09 | 0.01 | 0.08 | 0.08 | 0.18 | 0.09 | 0.10 |
| **18** | 0.01 | 0.02 | 0.01 | 0.00 | 0.01 | 0.02 | 0.00 | 0.00 | 0.00 | 0.03 | 0.09 | 0.01 | 0.08 | 0.12 | 0.01 | 0.11 | 0.07 | 0.01 | 0.06 |
| **19** | 0.38 | 0.24 | 0.13 | 0.03 | 0.02 | 0.24 | 0.09 | 0.13 | 0.00 | 0.00 | 0.06 | 0.07 | 0.22 | 0.09 | 0.05 | 0.09 | 0.07 | 0.12 | 0.09 |
| **20** | 0.07 | 0.03 | 0.06 | 0.03 | 0.07 | 0.03 | 0.01 | 0.06 | 0.09 | 0.01 | 0.06 | 0.08 | 0.19 | 0.13 | 0.09 | 0.09 | 0.16 | 0.09 | 0.08 |
| **21** | 0.08 | 0.00 | 0.00 | 0.00 | 0.08 | 0.00 | 0.04 | 0.00 | 0.01 | 0.02 | 0.14 | 0.11 | 0.23 | 0.11 | 0.10 | 0.10 | 0.15 | 0.01 | 0.18 |
| **22** | 0.05 | 0.00 | 0.18 | 0.11 | 0.05 | 0.00 | 0.04 | 0.09 | 0.06 | 0.13 | 0.01 | 0.09 | 0.04 | 0.01 | 0.01 | 0.01 | 0.15 | 0.11 | 0.14 |
| **X** | 0.02 | 0.09 | 0.01 | 0.09 | 0.01 | 0.09 | 0.08 | 0.32 | 0.06 | 0.29 | 0.04 | 0.01 | 0.00 | 0.02 | 0.02 | 0.02 | 0.06 | 0.09 | 0.09 |
| **Y** | 0.15 | 0.00 | 0.09 | 0.18 | 0.15 | 0.00 | 0.00 | 0.29 | 0.31 | 0.06 | 0.00 | 0.00 | 0.00 | 0.12 | 0.00 | 0.09 | 0.23 | 0.17 | 0.18 |

**Supplementary Table 3.** FISH Aneuploidy for Obstructive (OA) and Nonobstructive Azoospermic (NOA) Cohorts

| **Sample** | **chx13** | **chx15** | **chx16** | **chx17** | **chx18** | **chx21** | **chx22** | **XX** | **XY** | **YY** | **diploidy** | **nullisomy** | **Total Aneuploidy** |
| --- | --- | --- | --- | --- | --- | --- | --- | --- | --- | --- | --- | --- | --- |
| OA1 | 0.00 | 0.28 | 0.00 | 0.10 | 0.00 | 0.00 | 0.00 | 0.00 | 0.50 | 0.00 | 0 | 0 | 0.88 |
| OA2 | 0.11 | 0.11 | 0.00 | 0.28 | 0.40 | 0.11 | 0.11 | 0.00 | 0.10 | 0.00 | 0 | 0 | 1.22 |
| OA3 | 0.00 | 0.00 | 0.28 | 0.00 | 0.28 | 0.00 | 0.28 | 0.00 | 0.00 | 0.00 | 0 | 0 | 0.84 |
| OA4 | 0.10 | 0.20 | 0.20 | 0.20 | 0.10 | 0.50 | 0.00 | 0.30 | 0.00 | 0.00 | 0 | 0 | 1.6 |
| OA5 | 0.30 | 0.10 | 0.10 | 0.00 | 0.10 | 0.10 | 0.10 | 0.00 | 0.00 | 0.00 | 0 | 0 | 0.98 |
| OA6 | 0.28 | 0.14 | 0.00 | 0.14 | 0.28 | 0.14 | 0.14 | 0.10 | 0.00 | 0.00 | 0 | 0 | 1.22 |
| OA7 | 0.10 | 0.28 | 0.10 | 0.00 | 0.29 | 0.10 | 0.00 | 0.00 | 0.30 | 0.00 | 0 | 0 | 1.17 |
| OA8 | 0.30 | 0.00 | 0.00 | 0.50 | 0.10 | 0.30 | 0.00 | 0.10 | 0.10 | 0.00 | 0 | 0 | 1.6 |
| OA9 | 0.10 | 0.28 | 0.00 | 0.10 | 0.29 | 0.28 | 0.00 | 0.11 | 0.00 | 0.00 | 0 | 0 | 1.16 |
| OA10 | 0.00 | 0.28 | 0.00 | 0.27 | 0.20 | 0.00 | 0.00 | 0.00 | 0.20 | 0.10 | 0 | 0 | 1.05 |
| OA11 | 0.00 | 0.00 | 0.00 | 0.26 | 0.29 | 0.00 | 0.28 | 0.20 | 0.00 | 0.10 | 0 | 0 | 1.13 |
| OA12 | 0.00 | 0.20 | 0.00 | 0.26 | 0.14 | 0.20 | 0.00 | 0.10 | 0.20 | 0.20 | 0 | 0 | 1.3 |
| OA13 | 0.00 | 0.28 | 0.10 | 0.28 | 0.10 | 0.00 | 0.00 | 0.00 | 0.09 | 0.00 | 0 | 0 | 1.46 |
| OA14 | 0.25 | 0.00 | 0.22 | 0.28 | 0.26 | 0.00 | 0.00 | 0.09 | 0.00 | 0.00 | 0 | 0 | 1.1 |
| OA15 | 0.00 | 0.28 | 0.00 | 0.00 | 0.10 | 0.29 | 0.00 | 0.29 | 0.00 | 0.09 | 0 | 0 | 1.23 |
| OA16 | 0.00 | 0.00 | 0.10 | 0.00 | 0.14 | 0.26 | 0.00 | 0.09 | 0.09 | 0.00 | 0 | 0 | 1.13 |
| OA17 | 0.25 | 0.00 | 0.27 | 0.00 | 0.00 | 0.25 | 0.00 | 0.09 | 0.00 | 0.19 | 0 | 0 | 1.05 |
| OA18 | 0.00 | 0.29 | 0.00 | 0.29 | 0.00 | 0.29 | 0.19 | 0.00 | 0.00 | 0.09 | 0 | 0 | 1.15 |
| OA19 | 0.00 | 0.36 | 0.00 | 0.36 | 0.55 | 0.00 | 0.00 | 0.00 | 0.00 | 0.00 | 0 | 0 | 1.27 |
| NOA1 | 0.00 | 0.19 | 0.19 | 0.00 | 0.00 | 0.00 | 0.09 | 0.00 | 0.19 | 0.00 | 0 | 0 | 0.66 |
| NOA2 | 0.19 | 0.27 | 0.19 | 0.19 | 0.00 | 0.00 | 0.09 | 0.09 | 0.00 | 0.00 | 0 | 0 | 1.02 |
| NOA3 | 0.00 | 0.20 | 0.00 | 0.00 | 0.00 | 0.00 | 0.00 | 1.20 | 0.00 | 0.20 | 0 | 0 | 1.6 |
| NOA4 | 0.20 | 0.00 | 0.00 | 0.00 | 0.20 | 0.20 | 0.00 | 0.00 | 0.00 | 0.00 | 0 | 0 | 0.6 |
| NOA5 | 0.00 | 0.28 | 0.00 | 0.28 | 0.28 | 0.00 | 0.00 | 0.28 | 0.00 | 0.00 | 0 | 0 | 1.12 |
| NOA6 | 0.00 | 0.00 | 0.19 | 0.19 | 0.19 | 0.20 | 0.00 | 0.00 | 0.00 | 0.19 | 0 | 0 | 0.96 |
| NOA7 | 0.00 | 0.19 | 0.19 | 0.20 | 0.00 | 0.00 | 0.19 | 0.00 | 0.19 | 0.00 | 0 | 0 | 0.96 |
| NOA8 | 0.00 | 0.00 | 0.19 | 0.19 | 0.00 | 0.19 | 0.00 | 0.00 | 0.00 | 0.19 | 0 | 0 | 0.76 |
| NOA9 | 0.00 | 0.00 | 0.50 | 0.00 | 0.30 | 0.30 | 0.00 | 0.10 | 0.10 | 0.20 | 0 | 0 | 1.5 |
| NOA10 | 0.00 | 0.28 | 0.00 | 0.28 | 0.29 | 0.00 | 0.00 | 0.09 | 0.09 | 0.29 | 0 | 0 | 1.32 |
| NOA11 | 0.11 | 0.11 | 0.00 | 0.00 | 0.00 | 0.11 | 0.11 | 0.00 | 0.23 | 0.00 | 0 | 0 | 0.67 |

**Supplementary Table 4.** Localization, Type, and Minor Allele Frequency (MAF) of Main Mutations Identified in Study Participants as Compared to a Known Fertile Normozoospermic Control

| ***Obstructive*** | | | | | |  |
| --- | --- | --- | --- | --- | --- | --- |
| ***Gene*** | ***Chr*** | ***ID*** | ***Variant rs number*** | ***MAF*** | ***Pathogenicity Prediction*** | |
| *OR1D4* | 17 | ENSG00000255095 | rs2676568 | 0.76 | Unknown | |
| *SLC17A7* | 19 | ENSG00000104888 | rs565318583 | 0.69 | Unknown | |
| *ATP4A* | 19 | ENSG00000105675 | rs769485930 | 0.77 | Unknown | |
| ***Nonobstructive*** | | | | | |  |
| ***Gene*** | ***Chr*** | ***ID*** | ***Variant rs number*** | ***MAF*** | ***Pathogenicity Prediction*** | |
| *APOE* | 19 | ENSG00000130203 | rs758906237 | <0.01 | Unknown | |
| *AP1G2* | 14 | ENSG00000213983 | rs749982998 | 0.14 | Unknown | |
| *AP1S2* | X | ENSG00000182287 | rs757069069 | <0.01 | Unknown | |
| *AP5M1* | 14 | ENSG00000053770 | rs1189899 | 0.27 | Unknown | |
| *POLR2L* | 11 | ENSG00000177700 | rs34627090 | 0.72 | Unknown | |

**Supplementary Table 5.** Mutations Concurrent with Differentially Expressed Genes Identified in the Seminal Fluid of Nonobstructive Azoospermic Men

|  |  | **-Sperm** | |  | **+Sperm** | |
| --- | --- | --- | --- | --- | --- | --- |
| **Gene** | **Chr** | **Type** | **Location** |  | **Type** | **Location** |
| *NEU1* | 6 | Frameshift | c.48dup  c.402C>T  c.1072A>G  c.48dup  c.2056T>C |  | Synonymous | c.406G>A  c.1165C>T |
| *TPTE2* | 13 | Frameshift | c.1171C>T  c.2975G>A  c.2209C>T  c.772G>A |  | -- | -- |
| *TMCO5B* | 15 | -- | -- |  | -- | -- |
| *IGSF11-AS1* | 3 | Nonsense | c.331C>T  c.267C>T |  | Nonsense | c.272G>A  c.36C>T |
| *C10orf62* | 10 | Missense | c.363G>C  c.474A>G |  | Missense | c.363G>C  c.22A>G |
| *SMKR1* | 7 | Frameshift | c.21dup  n.50dup |  | -- | -- |
| *TRPC1* | 3 | Missense | c.2266G>A  c.1651T>C  c.1188G>C |  | -- | -- |
| *SPZ1* | 5 | Frameshift | c.618dup  c.25G>A  c.680A>G  c.986A>G  c.67C>T  c.131dup |  | -- | -- |

**Supplementary Table 6.** ICSI Clinical Outcomes for +Sperm Cohort Compared to a Normozoospermic Control Group

|  | **Control** | **+Sperm** |
| --- | --- | --- |
| **Couples** | 4 | 10 |
| **Maternal Age (M ± SD)** | 38.4±3 | 36.8±5 |
| **Paternal Age (M ± SD)** | 39.2±6 | 42.8±7 |
| **Cycles** | 4 | 26 |
| **Oocytes Retrieved** | 43 | 358 |
| **Fertilization (%)** | 27/35 (77.1) | 79/254 (31.1) |
| **Cycles with ET** | 4 | 21 |
| **Clinical Pregnancy (+FHB) (%)** | 4 (100) | 5 (23.8) |
| **Deliveries** | 4 | 3 |
